# Incidence, Risk Factors, and Mortality Associated With Orofacial Cleft Among Children in Ontario, Canada

**DOI:** 10.1001/jamanetworkopen.2019.21036

**Published:** 2020-02-12

**Authors:** Claudia C. Malic, Melody Lam, Jessy Donelle, Lucie Richard, Simone N. Vigod, Eric I. Benchimol

**Affiliations:** 1 Department of Surgery, University of Ottawa, Ottawa, Ontario, Canada; 2 Children’s Hospital of Eastern Ontario, Ottawa, Ontario, Canada; 3 ICES Western, London, Ontario, Canada; 4 ICES uOttawa, Ottawa, Ontario, Canada; 5 The Ottawa Hospital Research Institute, Ottawa, Ontario, Canada; 6 Division of Equity, Gender and Population, Department of Psychiatry, Faculty of Medicine, University of Toronto, Toronto, Ontario, Canada; 7 Women’s Mental Health Research, Women’s College Hospital and Research Institute, Toronto, Ontario, Canada; 8 ICES, Toronto, Ontario, Canada; 9 Department of Pediatrics, School of Epidemiology and Public Health, University of Ottawa, Ottawa, Ontario, Canada; 10 Health Information Technology Program, Children’s Hospital of Eastern Ontario Research Institute, Ottawa, Ontario, Canada

## Abstract

**Importance:**

Orofacial cleft (OFC) is one of the most common congenital malformations, with a wide variation in incidence worldwide. However, population-based studies on the incidence of OFC in North America are lacking.

**Objectives:**

To examine the incidence of OFC in Ontario, Canada, and to compare risk factors and mortality associated with children with OFC vs children without OFC.

**Design, Setting, and Participants:**

This population-based retrospective cohort study used health administrative data from the province of Ontario, Canada. Children with OFC who were born from April 1, 1994, to March 31, 2017, in Ontario were each matched to 5 children without OFC based on sex, date of birth (±30 days), and mother’s age (±5 years). Analyses were conducted from September 2018 to January 2019.

**Exposures:**

Children born with OFC.

**Main Outcomes and Measures:**

Incidence of OFC over time and regional variation. Risk factors for OFC were assessed using 1-way analysis of variance for means, Kruskal-Wallis for medians, and χ^2^ tests for categorical variables. Adjusted Cox regression models were used to assess mortality.

**Results:**

From 1994 to 2017, 3262 children were born with OFC in Ontario, Canada, and they were matched to 15 222 children born without OFC. Incidence of OFC in Ontario was 1.12 cases per 1000 live births, with wide geographic variation and a lower incidence from 2004 to 2017 compared with 1994 to 2003 (1.02 vs 1.13 cases per 1000 live births; *P* = .002), especially for the subgroup with cleft palate (0.52 vs 0.44 cases per 1000 live births; *P* = .006). Children with OFC, compared with children without OFC, were more likely to be born prematurely (406 children [13.3%] vs 1086 children [7.1%]; *P* < .001; standardized difference, 0.21) and had lower mean (SD) birth weight (3215.3 [687.6] g vs 3382.6 [580.0] g; *P* < .001; standardized difference, 0.26). The mortality rate among children with OFC was higher than among matched children without OFC (hazard ratio, 10.60; 95% CI, 7.79-14.44; *P* < .001). When mortality was adjusted for the presence of congenital or chromosomal anomalies, the risk of death was not significantly different between children with OFC and those without OFC (hazard ratio, 1.35; 95% CI, 0.73-2.72).

**Conclusions and Relevance:**

These findings suggest that incidence of OFC In Ontario, Canada, decreased from 1994 to 2017. Mortality in children with OFC was high, especially in the first 2 years of life, and was predominantly associated with the presence of other congenital or chromosomal anomalies. Further research is required to better understand the causes of wide geographical variations of OFC incidence and improve the survival of these patients.

## Key Points

**Question:**

What is the incidence of orofacial cleft (OFC) in Ontario, Canada, and what are the risk factors and mortality rate associated with OFC?

**Findings:**

This matched cohort study, including 3262 children with OFC in Ontario, Canada, matched with 15 222 children without OFC found that overall incidence of OFC declined from 1994 to 2017, and there was significant geographical variation. Compared with children without OFC, children with OFC were more likely to be born prematurely, and they had lower birth weight and a higher incidence of death in the first 2 years of life, especially when associated with other congenital or chromosomal anomalies.

**Meaning:**

These findings suggest that despite decreasing incidence of OFC, children with OFC should be monitored closely for adverse health outcomes.

## Teaser

This cohort study examines incidence of orofacial cleft among children in Ontario, Canada, and compares risks factors and mortality rates among children with vs without orofacial cleft.

## Introduction

Orofacial cleft (OFC) is one of the most common congenital malformations and includes 3 subgroups: cleft lip (CL), cleft palate (CP), and cleft lip and palate (CLP). The causes of OFC are complex, including genetic predisposition and environmental risk factors, but there is no evidence on the magnitude of risk contribution of these factors, either individually or in combination, to our knowledge.^1,2^ Racial/ethnic disparities have been observed in children with OFC.^2^ The incidence of OFCs is reported to be between 1 and 2 children with OFC per 1000 live births worldwide, with wide variability between regions, potentially owing to risk factors and cohort ascertainment methods. In Canada, 400 to 500 infants are born with OFC annually,^3,4^ with a reportedly stable incidence in the last few decades, despite public health efforts to reduce risk factors, such as encouraging perinatal folate intake, developing peripartum smoking cessation programs, and implementing earlier and more accurate prenatal screening.^5,6,7,8,9,10,11,12^ It is unclear if other sociodemographic or clinical factors, such as rural vs urban household, socioeconomic status, maternal history of diabetes, hypertension, epilepsy, mental health disorders, or adequacy of prenatal care are risk factors for OFC.^13^ Druschel et al^14^ reported that OFC associated with major congenital or chromosomal anomalies (known as *syndromic OFC*) was associated with increased infant mortality.^14,15,16^ Carlson et al^15^ reported a significant risk of mortality in the first year of life in children born with OFC. However, to our knowledge, there are no Canadian studies reporting mortality in children with OFC and its association with extrinsic risk factors.

Children with OFC are typically treated by a multidisciplinary team of health care practitioners throughout infancy and childhood and, as such, are likely to be high-cost health care users.^3^ In an era of patient-centered and preventive medicine, it is important to describe the incidence and characteristics of OFC and identify associated risk factors. The aims of this study were to determine the incidence of OFC and its subgroups in Ontario, Canada, and to identify risk factors and mortality associated with OFC compared with children without OFC.

## Methods

### Study Design and Setting

We conducted a retrospective matched cohort study of children born with OFC in Ontario, which is Canada’s most populous province (population, 14.4 million, as of April 2019),^17^ during a 23-year period, from April 1, 1994, to March 31, 2017 (fiscal years 1994-2016), using population-based health administrative data. During this period, Ontario’s single-payer universal health care system was administered through 14 Local Health Integration Networks (LHINs), which played an important role in the integration and coordination of services at the local level.^18^ Deidentified Ontario health administrative data are housed and available for research purposes at ICES (formerly Institute for Clinical Evaluative Sciences), an independent, nonprofit research institute funded by an annual grant from the Ontario Ministry of Health and Long-Term Care. ICES is an independent, nonprofit research institute whose legal status under Ontario’s health information privacy law allows it to collect and analyze health care and demographic data, without consent, for health system evaluation and improvement. Secure access to these data is governed by policies and procedures that are approved by the Information and Privacy Commissioner of Ontario. We had full access to the full, uncleaned data sets used for this study. The research ethics board of the Children’s Hospital of Eastern Ontario approved this study.

The reporting of this cohort study adhered to the Reporting of Studies Conducted Using Observational Routinely Collected Health Data (RECORD)^28^ and Strengthening the Reporting of Observational Studies in Epidemiology (STROBE) reporting guidelines. Data were analyzed from September 2018 to January 2019.

### Data Sources and Linkage

This study used information from the Canadian Institute for Health Information (CIHI) Discharge Abstract Database for hospitalization data, CIHI National Ambulatory Care Reporting System for emergency department care data, Ontario Health Insurance Plan (OHIP) for data on physician claims for outpatient services, ERCLAIMS for data on emergency department care derived from OHIP data, CIHI Same Day Surgery database for data on day procedures, Ontario Mental Health Reporting System for mental health diagnosis and care data, Registered Persons Database for data on sociodemographic characteristics, and Mother-Baby Linked Database, which links maternal and neonate records through the CIHI Discharge Abstract Database, for data on all in-hospital births (>98% of the population) (eTable 1 in the Supplement). Individual-level records were linked across databases using encrypted ICES unique identification numbers derived from provincial health card numbers. All residents of Ontario who have a valid health card and are eligible for universal health care are contained within these databases (>99% of the population). For CIHI databases, diagnosis and procedural codes followed the *International Classification of Diseases, Ninth Revision* (*ICD-9*)^19^ and *Canadian Classification of Diagnostic, Therapeutic and Surgical Procedures* (*CCP*) prior to April 1, 2002; thereafter, we used the Canadian version of the *International Statistical Classification of Diseases and Related Health Problems, Tenth Revision* (*ICD-10-CA*) and *Canadian Classification of Health Interventions* (*CCI*).^20^ For OHIP databases, a simplified 3-digit version of *ICD-9* was linked to each claim. Diagnosis codes for mental disorders in the Ontario Mental Health Reporting System followed the *Diagnostic and Statistical Manual of Mental Disorders* (Fourth Edition).^21^

### Study Population

We included all children born in Ontario hospitals from April 1, 1994, to March 31, 2017. The cohort entry date was the child’s birthdate. After applying the initial exclusion criteria, we created 2 cohorts (hereafter, *initial cohort* and *final cohort*) (eFigure in the Supplement). The initial cohort was used to describe OFC incidence and included all children diagnosed with OFC within the first 30 days of life and all children who never received a diagnosis of OFC (unexposed group).

The final cohort further excluded children ineligible for Ontario health care at any point during the study and children whose mothers were not eligible for Ontario health care for at least 1 year prior to their child’s birth. Each child with OFC was matched with 5 children without OFC based on sex, date of birth (±30 days), and mother’s age (±5 years). Children for whom matches could not be found were excluded. This final cohort was used to determine mortality and assess risk factors.

### Exposure

We identified OFC and subgroup diagnoses with *ICD-9* codes 749.0, 749.1, and 749.2 and *ICD-10-CA* diagnostic codes Q35.x, Q36.x, and Q37.x, which have been used in previous studies^3,4^ but have not been validated in Ontario. eTable 2 in the Supplement describes the codes used to classify children with CL, CP, or CLP. A child with OFC who was also diagnosed in the first 30 days after birth with a diagnosis of congenital or chromosomal anomalies was considered to have syndromic OFC (eTable 2 in the Supplement). When diagnosis codes and procedural codes (*CCI*, *CCP*, and OHIP fee codes) for a cleft surgical procedure within 2 years after birth indicated different subgroup classifications, the precedence was given to the type of cleft surgical procedure that was performed.

### Variables of Interest

Variables assessed included sex, Rurality Index of Ontario (RIO) score,^22^ mean neighborhood income quintile (a validated proxy for individual-level income^23^), LHIN, prematurity status, birth weight, and type of surgical procedure performed in the first 2 years of life (eTable 2 in the Supplement). The RIO score is a reflection of rurality that depends on geographical factors, including population density, as well as health services factors, such as travel time to basic and advanced referral centers.^24^ Scores range from 0 to 100, with higher scores indicating more rurality.

Maternal characteristics included age at the child’s birth, number of live births in the past 2 years, type and number of prenatal care visits, the presence of comorbidities (ie, hypertension, diabetes, and epilepsy) in the 2 years prior to the child’s birth, and history of mental health–related care within 3 years prior to the child’s birth (eTable 2 in the Supplement). Classification of the mother’s mental health–related care used the Steele categorization.^25^ Additionally, gestational diabetes, gestational hypertension, labor induction, use of instrumentation, and cesarean delivery were determined. Any diagnoses of hypertension or diabetes from 120 days prior to the child’s birth to 180 days after the child’s birth were considered gestational.^26,27^ Additionally, we determined and compared the rate of and time to death of any cause in the OFC and non-OFC groups.

### Statistical Analysis

Using the initial cohort, we calculated the crude incidence of OFC and its subgroups per 1000 live births. We used 2-sample *t* tests to compare incidence across 2 periods (1994-2003 vs 2004-2017), and 1-sample *t* tests to compare incidence across LHINs for these 2 periods.

Using the final cohort, we summarized the child and maternal characteristics using means and SDs for normally distributed data or medians and interquartile ranges for nonnormally distributed data, with comparisons between OFC and non-OFC groups using 1-way analysis of variance for means, Kruskal-Wallis test for medians, and χ^2^ test for categorical variables. *P* values were 2-sided, and *P* < .05 was considered statistically significant. Weighted standardized differences were calculated to assess significance while taking the sample size into account. Adjusted Cox proportional hazards regression models were used to compare mortality in the OFC group and its subgroups to the matched non-OFC group and also to identify risk factors for OFC. We used SAS statistical software version 9.4 (SAS Institute) for all statistical analyses. All cell sizes comprising fewer than 6 people were suppressed to adhere to privacy regulations.

## Results

### Incidence and Variation of OFC

**Figure 1.**
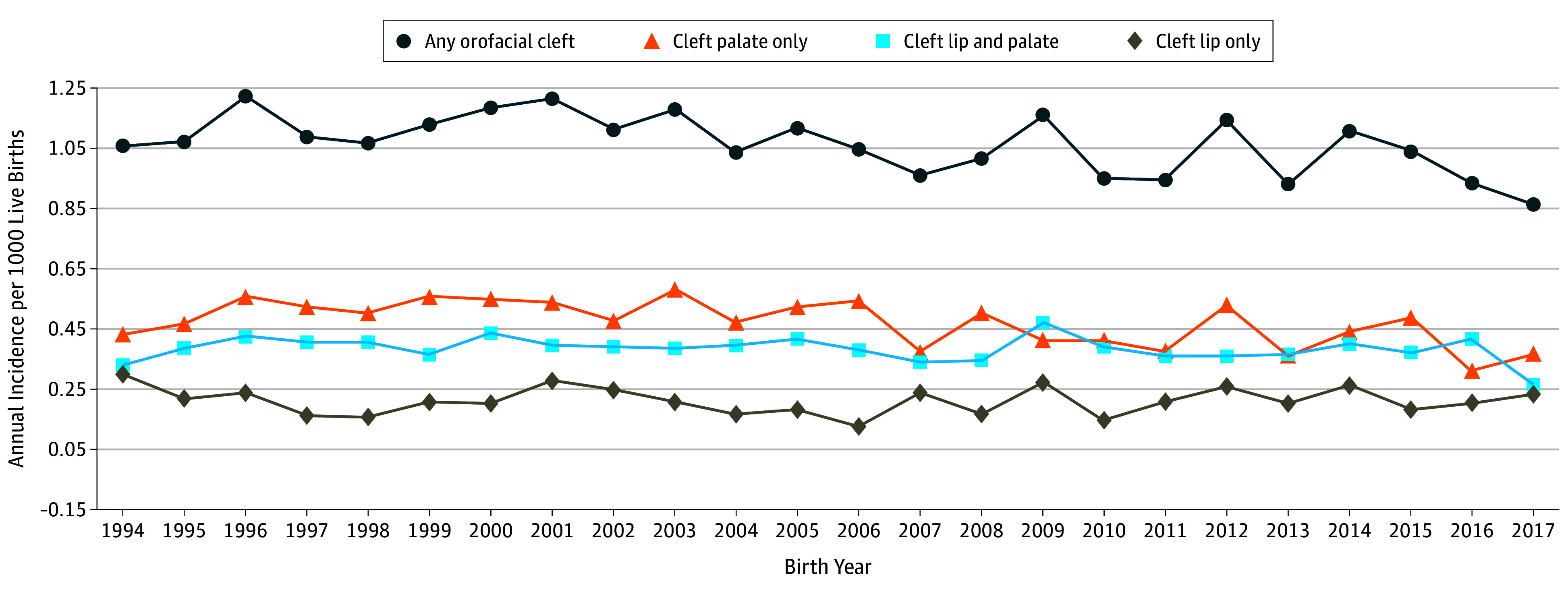
Incidence of Orofacial Cleft per 1000 Live Births Between April 1, 1994, and March 31, 2017

**Figure 2.**
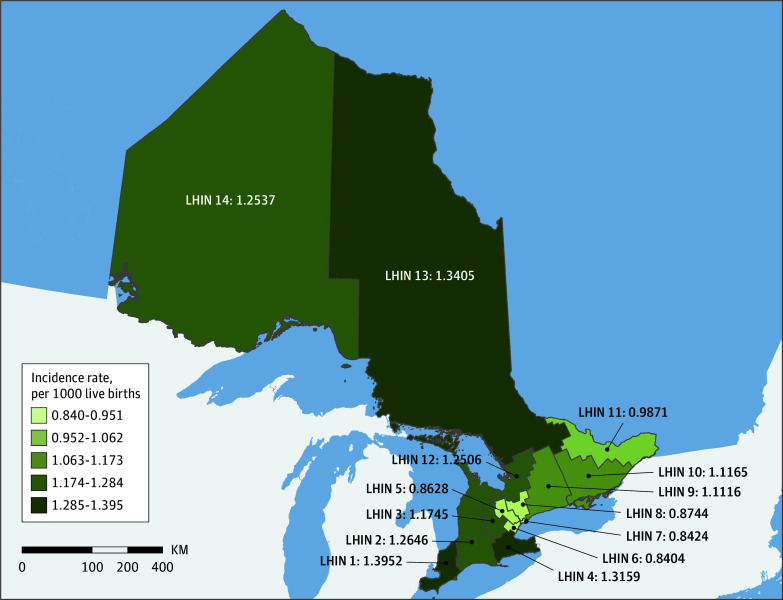
Incidence of Orofacial Cleft per 1000 Live Births Across 14 Local Health Integration Networks (LHINs) in Ontario, Canada, Between April 1, 1994, and March 31, 2017 LHIN 1 indicates Erie St Clair; 2, South West; 3, Waterloo Wellington; 4, Hamilton Niagara Haldimand Brant; 5, Central West; 6, Mississauga Halton; 7, Toronto Central; 8, Central; 9, Central East; 10, South East; 11, Champlain; 12, North Simcoe Muskoka; 13, North East; and 14, North West.

A total of 3262 children were included in the OFC group from a total of 2 923 852 births for the incidence calculation (eFigure in the Supplement). The overall incidence of OFC was 1.12 cases per 1000 live births, with incidence ranging over time from 1.22 cases per 1000 live births in 1996 to 0.86 cases per 1000 live births in 2017 (Figure 1). Incidence significantly decreased from 1.13 cases per 1000 live births in the 1994 to 2003 period to 1.02 cases per 1000 live births in the 2004 to 2017 period (*P* = .002). Incidence also decreased in the CP subgroup (0.52 vs 0.44 cases per 1000 live births; *P* = .006). Incidence varied geographically across LHINs, with lower incidence recorded in the Central West, Mississauga Halton, Toronto Central, and Central LHINs, representing the Greater Toronto Area, the largest urban center in Ontario (Figure 2; eTable 3 in the Supplement).

### Characteristics of Included Children

The final cohort included 3051 children with OFC (1344 girls [44.1%]) and 15 255 matched children without OFC (eFigure in the Supplement). Among subtypes, 1345 children (44.1%) had CP, 1111 children (36.4%) had CLP, and 595 children (19.5%) had CL. Children from the CP subgroup underwent palate repair later than the children from CLP subgroup (mean [SD] time to palate repair, 396.3 [87.4] days vs 346.6 [118.8] days; *P* < .001). The male to female ratios were higher in the CL (1.95:1) and CLP (1.87:1) subgroups (eTable 4 in the Supplement).

A greater proportion of children with OFC were born prematurely (gestational age <37 weeks) compared with children without OFC (406 children [13.3%] vs 1086 children [7.1%]; *P* < .001; standardized difference, 0.21). Mean (SD) weight at birth was significantly lower among children with OFC than children without OFC (3215.3 [687.6] g vs 3382.6 [580.0] g; *P* < .001; standardized difference, 0.26), even when analysis was restricted to children born at term (3369.3 [547.1] g vs 3463.1 [486.2] g; *P* < .001; standardized difference, 0.18) or children born prematurely (2210.8 [666.3] g vs 2331.8 [673.1] g; *P* = .002; standardized difference, 0.18) (Table 1).

**Table 1.** Descriptive Characteristics of Final Cohort

Characteristic	No. (%)		Standardized Difference	*P* Value
	OFC (n = 3051)	Non-OFC (n = 15 255)		
Residence Rurality Index of Ontario score^a^				
0	608 (19.9)	3460 (22.7)	0.07	.003
1 to <40	2063 (67.6)	10 117 (66.3)	0.03	
≥40	336 (11.0)	1495 (9.8)	0.04	
Missing	44 (1.4)	183 (1.2)	0.02	
Neighborhood income quintile				
1 (Lowest)	693 (22.7)	3354 (22.0)	0.02	.11
2	675 (22.1)	3100 (20.3)	0.04	
3	608 (19.9)	3093 (20.3)	0.01	
4	580 (19.0)	3154 (20.7)	0.04	
5 (Highest)	480 (15.7)	2487 (16.3)	0.02	
Missing	15 (0.5)	67 (0.4)	0.01	
Premature birth^b^	406 (13.3)	1086 (7.1)	0.21	<.001
Weight at birth, g				
All children				
Mean (SD)	3215.3 (687.6)	3382.6 (580.0)	0.26	<.001
Median (IQR)	3265 (2840-3670)	3410 (3060-3745)	0.24	<.001
Children born at term				
Mean (SD)	3369.3 (547.1)	3463.1 (486.2)	0.18	<.001
Median (IQR)	3368 (3000-3740)	3450 (3130-3775)	0.17	<.001
Children born preterm				
Mean (SD)	2210.8 (666.3)	2331.8 (673.1)	0.18	.002
Median (IQR)	2270 (1735-2625)	2380 (1950-2760)	0.19	.001

Abbreviations: IQR, interquartile range; OFC, orofacial cleft.

^a^ Higher score (range, 0-100) indicates increased rurality.

^b^ Defined as birth at less than gestational age 37 weeks.

Although a large proportion of children with OFC (2671 children [87.5%]) lived in urban areas (RIO score, 0-40), children with OFC were more likely to live in rural areas (RIO score, >40) than children without OFC (336 children [11.0%] vs 1495 children [9.8%]; *P* = .003; standardized difference, 0.04). There was no significant difference in income distribution between the 2 cohorts (*P* = .11; standardized difference, 0.02).

### Maternal Characteristics

Mothers of children with OFC had a higher prevalence of epilepsy within the past 2 years (32 women [1.0%] vs 85 women [0.6%]; *P* = .002; standardized difference, 0.06) and having received mental health care within the past 3 years (132 women [4.3%] vs 503 women [3.3%]; *P* = .005; standardized difference, 0.05) compared with mothers of children without OFC (Table 2). Rates of hypertension, diabetes, gestational hypertension, and gestational diabetes were similar in both groups. Mothers of children with OFC were more likely to undergo a cesarean delivery than mothers of children without OFC (954 women [31.3%] vs 3927 women [26.0%]; *P* < .001; standardized difference, 0.12) and had fewer mean (SD) prenatal visits (9.2 [3.8] visits vs 9.5 [3.7] visits; *P* < .001; standardized difference, 0.09).

**Table 2.** Maternal Characteristics of Final Cohort

Characteristic	No. (%)		Standardized Difference	*P* Value
	OFC (N = 3051)	Non-OFC (N = 15 255)		
Age, y				
Mean (SD)	29.6 (5.7)	29.6 (5.5)	0	.98
≤20	200 (6.6)	980 (6.4)	0	.98
21-30	1485 (48.7)	7443 (48.8)	0	.17
31-40	1295 (42.4)	6566 (43.0)	0.01	
≥41	71 (2.3)	266 (1.7)	0.04	
Adequate prenatal care visits^a^	2750 (90.1)	13 894 (91.1)	0.03	.10
Type of prenatal care^b^				
>75% General practitioner or family physician	517 (18.8)	2894 (20.8)	0.05	.02
>75% with OBGYN (>75%)	1561 (56.8)	7845 (56.5)	0.01	
Both but <75% of visits each	672 (24.4)	3155 (22.7)	0.04	
Prenatal care visits, mean (SD), No.^b^				
Overall	9.2 (3.8)	9.5 (3.7)	0.09	<.001
Distribution by LHIN				
Erie St Clair	9.1 (3.7)	9.6 (3.5)	0.13	.11
South West	9.2 (3.6)	9.2 (3.8)	0	>.99
Waterloo Wellington	8.6 (4.3)	9.0 (4.3)	0.09	.27
Hamilton Niagara Haldimand Brant	9.1 (4.0)	9.5 (4.0)	0.09	.10
Central West	9.4 (3.5)	9.6 (3.3)	0.05	.48
Mississauga Halton	9.7 (3.4)	9.9 (3.1)	0.08	.24
Toronto Central	9.4 (3.4)	9.5 (3.8)	0	.96
Central	9.9 (3.2)	10 (3.2)	0.03	.60
Central East	9.7 (3.6)	9.8 (3.5)	0.04	.53
South East	7.7 (4.0)	8.2 (4.2)	0.13	.25
Champlain	9.1 (3.8)	9.8 (3.5)	0.19	.01
North Simcoe Muskoka	8.9 (3.9)	9.4 (3.7)	0.13	.23
North East	7.7 (4.1)	8.3 (4.4)	0.14	.12
North West	7.5 (4.4)	7.1 (4.7)	0.09	.50
Maternal live births in the previous 2 y				
0	2668 (87.4)	13293 (87.1)	0.01	.03
1	373 (12.2)	1943 (12.7)	0.02	
2	10 (0.3)	19 (0.1)	0.04	
≥3	0	0	0	
Mother received mental health care within 3 y	132 (4.3)	503 (3.3)	0.05	.005
Maternal comorbidities within 2 y				
Diabetes	17 (0.6)	86 (0.6)	0	.97
Hypertension	19 (0.6)	91 (0.6)	0	.86
Epilepsy	32 (1.0)	85 (0.6)	0.06	.002
Pregnancy complications				
Gestational diabetes 120 d before to 180 d after index date	36 (1.2)	129 (0.8)	0.03	.07
Gestational hypertension 120 d before to 180 d after index date	175 (5.7)	805 (5.3)	0.02	.30

Abbreviations: LHIN, Local Health Integration Network; OBGYN, obstetrician or gynecologist; OFC, orofacial cleft.

^a^ Defined as more than 3 visits.

^b^ Restricted to mothers who had more than 3 visits.

### Mortality

Mortality in the OFC group was significantly higher than in the non-OFC group (5.08 vs 0.33 deaths per 1000 person-years [PYs]; *P* < .001), with significantly lower mean (SD) time to death among those who died (1.3 [3.0] years vs 3.4 [6.1] years; *P* < .001; standardized difference, 0.45), especially in the CLP subgroup (0.9 [3.0] years). The death rate in the CP subgroup was 6.09 deaths per 1000 PYs, compared with 5.55 deaths per 1000 PYs in the CLP subgroup and 1.95 deaths per 1000 PYs in the CL subgroup. After the initial increase of death rate in the first 2 years of life in the OFC group, the risk of death was similar to the non-OFC group thereafter. After adjustment for all child and maternal characteristics, the risk of death was higher in the OFC group compared with the non-OFC group (hazard ratio [HR], 10.60; 95% CI, 7.79-14.44; *P* < .001). When mortality was adjusted for the presence of congenital or chromosomal anomalies (ie, syndromic OFC), the hazard was not statistically significantly different between OFC and non-OFC groups (HR, 1.36; 95% CI, 0.73-2.72). When OFC subgroups were included in the model and adjusted for syndromic subgroup, the hazard of death was highest in the CLP group compared with the non-OFC group (HR, 1.99; 95% CI, 1.06-3.73; *P* = .03). Factors that were significantly associated with an increased risk of mortality in the multivariable regression model were lower weight at birth (HR per 1-g increase, 0.99; 95% CI, 0.99-0.99; *P* < .001), born in the North East LHIN (HR, 2.31; 95% CI, 1.28-4.19; *P* = .005), presence of other congenital or chromosomal anomalies (HR, 24.85; 95% CI, 13.80-44.73; *P* < .001), having a mother who had 1 other live birth in the previous 2 years (HR, 2.89; 95% CI, 0.99-8.50; *P* < .001), fewer prenatal care visits (HR per 1-unit increase in prenatal visits, 0.94; 95% CI, 0.91-0.97; *P* < .001), and labor induction (HR, 1.73; 95% CI, 1.34-2.23; *P* < .001) (Table 3). Being born in the Hamilton Niagara Haldimand Brant LHIN was associated with lower risk of mortality (HR, 0.53; 95% CI, 0.30-0.91; *P* = .02), as was gestational hypertension within 280 days prior to birth (HR, 0.57; 95% CI, 0.35-0.93; *P* = .03). There were inconsistent associations of neighborhood income quintile with mortality, as income in the first (lowest) quintile (HR, 2.35; 95% CI, 1.50-3.67; *P* < .001), third quintile (HR, 1.95; 95% CI, 1.23-3.09; *P* = .004), and fourth quintile (second highest) (HR, 2.30; 95% CI, 1.46-3.63; *P* < .001) were associated with significant higher mortality risk. The multivariate logistic regression model found that mothers who lived in the North West (odds ratio [OR] 1.69; 95% CI, 1.27-2.24) or North East (OR, 2.01; 95% CI, 1.38-2.94) LHIN, were treated for epilepsy (OR, 1.72; 95% CI, 1.04-2.89), or who had 2 previous live births in the preceding 2 years (OR, 2.76; 95% CI, 1.06-7.20) had a higher risk of having a child with OFC.

**Table 3.** Adjusted Hazard Ratios for Mortality

Variable	Adjusted Hazard Ratio (95% CI)	*P* Value
OFC		
None	1 [Reference]	NA
Any	10.60 (7.79-14.44)^a^	<.001
Lip	0.63 (0.29-1.36)	.23
Palate	1.99 (1.06-3.73)	.03
Lip and palate	1.08 (0.57-2.05)	.82
Maternal age at delivery, per 1-y increase	1.00 (0.98-1.02)	.87
Sex		
Female	1 [Reference]	NA
Male	0.76 (0.60-0.95)	.01
Rurality Index of Ontario score, per 1-unit increase	1.00 (0.99-1.01)	.59
Neighborhood income quintile		
1 (Lowest)	2.35 (1.50-3.67)	<.001
2	1.46 (0.90-2.35)	.12
3	1.95 (1.23-3.09)	.004
4	2.30 (1.46-3.63)	<.001
5 (Highest)	1 [Reference]	NA
Missing	3.43 (0.45-26.22)	.23
Preterm birth^b^	1.04 (0.74-1.44)	.83
Weight at birth, per 1-g increase	0.99 (0.99-0.99)	<.001
Live births in the previous 2 y	1.63 (1.20-2.21)	.002
0	1 [Reference]	NA
1	2.89 (0.99-8.50)	.05
2	0.94 (0.91-0.97)	<.001
No. of prenatal care visits, per 1-unit increase	0.94 (0.91-0.97)	<.001
Maternal comorbidity^c^		
Psychiatric disorder	1.28 (0.75-2.17)	.36
Diabetes	0.72 (0.22-2.36)	.58
Hypertension	0 (0-5.20)	.96
Epilepsy	0.84 (0.32-2.19)	.71
Gestational diabetes in the 120 d previous and 180 d after index date	1.51 (0.73-3.14)	.26
Gestational hypertension in the 280 d previous index date	0.57 (0.35-0.93)	.03
Labor induction	1.73 (1.34-2.23)	<.001
Instrumentation needed	0.61 (0.39-0.96)	.04
Cesarean delivery	1.26 (0.99-1.61)	.06
Syndromic indicator	24.85 (13.80-44.73)	<.001
Year of birth, per 1-y increase	0.97 (0.95-0.99)	.005
Lowest annual rate per LHIN		
Erie St Clair	0.72 (0.39-1.32)	.29
South West	0.94 (0.54-1.64)	.83
Waterloo Wellington	0.83 (0.45-1.53)	.56
Hamilton Niagara Haldimand Brant	0.53 (0.30-0.91)	.02
Central West	0.52 (0.27-1.03)	.06
Mississauga Halton	1.08 (0.63-1.85)	.78
Toronto Central	0.88 (0.50-1.51)	.64
Central	0.94 (0.56-1.55)	.79
Central East	0.68 (0.34-1.37)	.28
South East	0.69 (0.39-1.20)	.19
Champlain	1.10 (0.55-2.21)	.78
North Simcoe Muskoka	2.31 (1.28-4.19)	.005
North East	0.96 (0.37-2.48)	.93

Abbreviations: LHIN, Local Health Integration Network; NA, not applicable; OFC, orofacial cleft.

^a^ Derived from the Cox proportional hazards model and adjusted for predetermined baseline covariates, including age, sex, subgroup of OFC (ie, cleft lip, cleft palate, or cleft lip and palate), maternal mental health care history, maternal diabetes, maternal age at birth, weight at birth, maternal preeclampsia, maternal gestational diabetes, year of birth, presence of other congenital and chromosomal anomalies (syndromic indicator), and LHIN.

^b^ Birth at term used as the reference value.

^c^ Children of mothers without the given comorbidity were used as the reference.

## Discussion

In this population-based cohort study of children with OFC from Ontario, Canada, we observed a lower incidence of OFC than previously reported in Canada^4^ and decreasing incidence from 1994 to 2017. There was large geographical variation of incidence of OFC across Ontario. Prematurity and low birth weight were more prevalent in children with OFC, especially in the CP subgroup. Risk factors associated with OFC included rurality, maternal history of epilepsy, and maternal history of mental health care. There was also a higher mortality rate in children with OFC, especially in the first 2 years of life, particularly associated with the presence of congenital and chromosomal anomalies diagnosed in the first 30 days of life.

### Incidence of OFC and Its Variation

Previous Canadian data indicate that the incidence of OFC across Canada was stable during the last 3 decades.^3,4^ The Canadian Congenital Anomalies Surveillance System reported a prevalence for OFC of 1.46 cases per 1000 total live births and stillbirths for Ontario from 1998 to 2007, with a slight decline of CL and CLP rates.^29^ Our reported incidence of OFC and its subgroups was lower than previously reported across Canada, and to our knowledge, this is the first study to report that the incidences of OFC and CP are declining. A 2014 study by Lowry et al^30^ reported a slightly increased rate of CL and CLP but a significant decrease of CP. Comparisons of OFC incidence across studies are difficult, as there are differences in the composition of the cohorts, such as racial and ethnic differences, whether syndromic and nonsyndromic OFC are included, differences in categorization (CLP and CP subgroups are often grouped together), and whether stillbirths and terminations are captured.^30^ Fluctuations are commonly seen in epidemiological studies describing congenital anomalies, but it is rare to see significant changes in incidence unless a significant intervention to prevent the disease has occurred. In addition, population health interventions, such as folate supplementation, have been shown to have no or minimal effect in previous studies in Canada,^29^ the United States, and Chile.^31^

The lower OFC incidence reported in some regions of Ontario (particularly the Greater Toronto area) may be associated with the unique demographic characteristics of those areas. Rural residence at birth was found to be a risk factor for OFC, potentially related to access to prenatal care. Rural residence, lower income, and family physician–delivered prenatal care have been associated with low screening rates in the first trimester of pregnancy in the general population.^18^ As CL and CLP can be diagnosed antenatally with ultrasonographic screening, higher screening rates and more prenatal care in urban populations may have resulted in more frequent therapeutic abortions, potentially explaining lower incidence of OFC in large urban centers. Additionally, different beliefs to continue a pregnancy if fetal anomalies are noted may influence incidence of OFC. Further research is needed to understand if there is an association between access to care and regional variation in incidence of OFC across Ontario, especially in rural areas.

### Characteristics of Children Born With OFC

Children with OFC had lower birth weight and higher prevalence of prematurity than the non-OFC group, which is consistent with a 2013 study by Pavri and Forrest.^3^ Certain maternal risk factors (eg, obesity, smoking, alcohol intake) have also been identified, but their association with OFC was inconclusive.^2,13,32^

Socioeconomic status has been reported in the literature as a potential risk factor for OFC and likely reflects differential use of the health system and poor access to prenatal education and care. Socioeconomic status is measured with a variety of indicators, including education, income, and occupation. Previous UK studies^33,34^ indicated a linear correlation between the level of deprivation and the risk of having a child with OFC, which was mediated by higher incidence of smoking during pregnancy. A 2009 study from California^35^ indicated that low socioeconomic status was associated with increased risk of having a child with OFC. Our study did not demonstrate an association between neighborhood income quintile and risk of OFC, consistent with a 2016 Norwegian study.^36^ It is possible that income is too crude a measure of socioeconomic status or the effect of social deprivation is blunted in the context of Ontario’s universal health care system.

### Characteristics of Mothers

Most maternal risk factors identified previously were not found to be associated with OFC in this study, concurring with a study by Raut et al.^13^ The association of maternal history of mental health–related care with OFC is novel. However, our finding that maternal epilepsy is a risk factor associated with OFC is consistent with the literature.^37,38,39^ Further research is required to determine the specific maternal psychiatric disorders that are associated with OFC. Alternatively, medications taken for certain psychiatric conditions may increase the risk factor for OFC.

### Mortality

A 2013 meta-analysis^15^ suggested that the presence of congenital or chromosomal anomalies was associated with a higher rate of infant mortality in children with OFC, which could explain the high mortality and young age at death in the CP and CLP subgroups in our cohort. The increased risk of mortality in our study was attributed to the presence of syndromic OFC, as children with nonsyndromic OFC had similar mortality risk as children without OFC. To our knowledge, this is the first Canadian study to report the mortality of children with OFC. A 2004 study by Christensen et al^40^ indicated that the most frequent causes of death for children with CLP and CP were prematurity, pneumonia, bronchopneumonia, and undiagnosed brain anomalies. Future exploration of risk factors of death could improve the survival of these children.

### Strength and Limitations

This study has some strengths, including its large sample size and its population-based nature. Additionally, owing to the use of health administrative data, which allowed deterministic and probabilistic linkage between mother and child, we were able to assess maternal risk factors up to 2 years before birth.

This study also has some limitations. As with all research using health administrative data, there is the risk of misclassification bias.^28^ Our method of identification of patients with OFC has not been validated; however, the diagnostic codes associated with hospitalization data in Canada have been demonstrated to be accurate for a variety of other conditions that require surgical procedures.^41^ We aimed to reduce errors in OFC diagnostic subtypes by verifying them with the procedural codes for cleft surgery performed in the first 2 years of life. Another potential bias is that we did not take into account any diagnosis of congenital or chromosomal anomalies after 30 days from birth, as the youngest children in the OFC cohort were aged 30 days. Had we classified children who were diagnosed with congenital or chromosomal anomalies after age 30 days as syndromic, the mortality estimates would have been subject to the risk of immortal time bias, since some syndromic children may have died after age 30 days but before their official diagnosis as syndromic. Owing to missing variables in our health administrative data, we were unable to link the geographical variation in the incidence of OFC with other confounders, such as smoking during pregnancy and higher incidence of psychiatric disorders in certain areas of Ontario. We did not have access to reliable medication data for patients who were not using social assistance programs, so we could not assess if psychiatric diagnoses or the medications used to treat these diagnoses were associated with OFC.

Some of the geographic variation in incidence may have resulted from geographic or cultural differences in the acceptance of or access to therapeutic abortions. Unfortunately, these variables were not available in the data sets. Additionally, although OFC was associated with a higher risk of death in our population, as with all observational studies, this association may not be causative.

## Conclusions

The findings of this cohort study suggest that incidence of OFC in Ontario is lower than previously reported in Canadian studies, with large geographical variations. There was decreased incidence over time, especially for the CP subgroup. Children with OFC were more likely to be born prematurely and had lower birth weight than children without OFC. Maternal epilepsy and receipt of mental health–related care were identified as risk factors of OFC. Mortality in children with OFC was significantly higher in the first 2 years of life than in children without OFC, especially in children with CP or CLP. Further research is required to identify factors associated with high mortality among children born with OFC.

## Notes

Supplement. **eFigure.** Inclusion and Exclusion Criteria for Cohorts of OFC and Non-OFC Groups **eTable 1.** Health Administrative Databases Used for This Study **eTable 2.** Diagnostic and Procedural Codes Used in This Study **eTable 3.** Comparison of Overall OFC Incidence From FY 1994 to 2017 With the Decade FY 2007-2016 Within Each LHIN **eTable 4.** Distribution and Timing of Surgical Procedures for Cleft Lip and/or Cleft Palate Repair in the OFC Cohort
