# Supplementary material for: Incidence, Risk Factors, and Mortality Associated With Orofacial Cleft Among Children in Ontario, Canada
Source: JAMA Netw Open. 2020 Feb 12;3(2):e1921036. doi: 10.1001/jamanetworkopen.2019.21036 (PMC11849746; doi:10.1001/jamanetworkopen.2019.21036)
Supplement: Supplement. — eFigure. Inclusion and Exclusion Criteria for Cohorts of OFC and Non-OFC Groups eTable 1. Health Administrative Databases Used for This Study eTable 2. Diagnostic and Procedural Codes Used in This Study eTable 3. Comparison of Overall OFC Incidence From FY 1994 to 2017 With the Decade FY 2007-2016 Within Each LHIN eTable 4. Distribution and Timing of Surgical Procedures for Cleft Lip and/or Cleft Palate Repair in the OFC Cohort [file jamanetwopen-e1921036-s001.pdf]

# Supplementary Online Content

Malic CC, Lam M, Donelle J, Richard L, Vigod SN, Benchimol EI. Incidence, risk factors, and mortality associated with orofacial cleft among children in Ontario, Canada. *JAMA Netw Open*. 2020;3(2):e1921036. doi:10.1001/jamanetworkopen.2019.21036

**eFigure.** Inclusion and Exclusion Criteria for Cohorts of OFC and Non-OFC Groups
**eTable 1.** Health Administrative Databases Used for This Study
**eTable 2.** Diagnostic and Procedural Codes Used in This Study
**eTable 3.** Comparison of Overall OFC Incidence From FY 1994 to 2017 With the Decade FY 2007-2016 Within Each LHIN
**eTable 4.** Distribution and Timing of Surgical Procedures for Cleft Lip and/or Cleft Palate Repair in the OFC Cohort

This supplementary material has been provided by the authors to give readers additional information about their work.

© 2020 Malic CC et al. *JAMA Network Open.*

**eFigure.** Inclusion and Exclusion Criteria for Cohorts of OFC and Non-OFC Groups

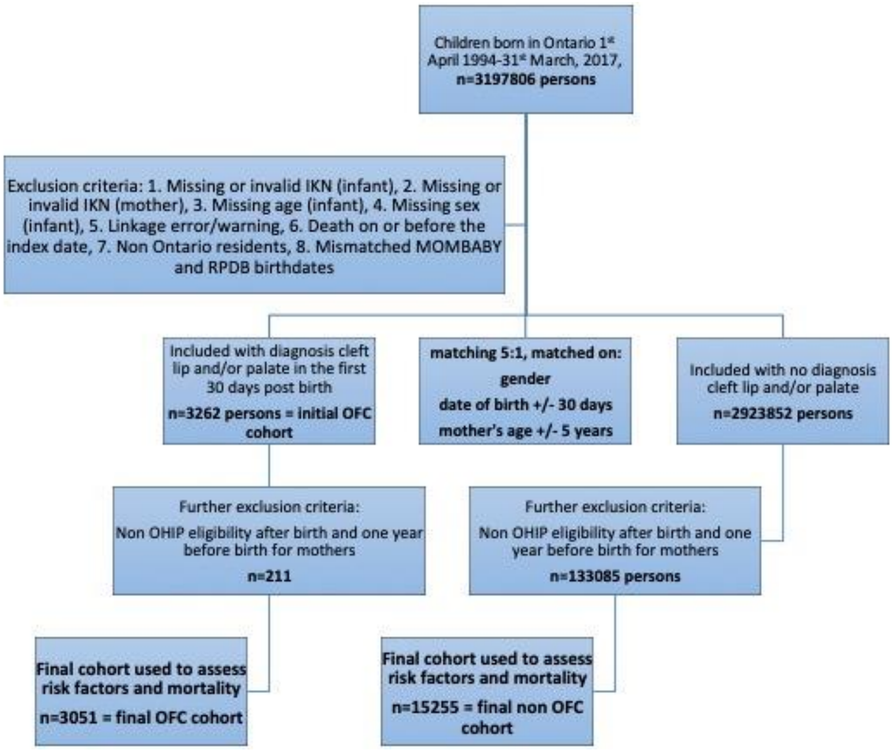


© 2020 Malic CC et al. *JAMA Network Open.*

**eTable 1.** Health Administrative Databases Used for This Study

| **Abbreviation** | **Health Administrative Database** | **Description** |
|---|---|---|
| CIHI DAD | Discharge Abstract Database | Medical hospitalization for mother and child and psychiatric hospitalizations for mother 1993-2017 |
| NACRS | National Ambulatory Care Reporting System | Emergency department visits for mother one year before and during the pregnancy |
| OHIP | Ontario Health Insurance Plan Claims Database | Outpatients physician billing data |
| IPDB | ICES Physician Database | Specialty of the physicians |
| OMHRS | Ontario Mental Health Reporting System | Psychiatric hospitalization for mothers one year before birth |
| CIHI SDS | Same Day Surgery Database (Annual) | Cleft surgeries performed in the first 2 years of life |
| CONTACT | Yearly Health Services Contact | Yearly health services contact |
| ERCLAIM | OHIP's Emergency Claims Database | Emergency billing claims for mothers pre- and perinatal care |
| RPDB | Registered Persons Database | Information about all residents with health cards in Ontario |
| CENSUS | Ontario Census Area Profiles | Population and demographics in Ontario |
| LHIN | Local Health Integration Network | Information about LHIN structure, geographical regions |
| PCCF | Postal Code Conversion File | Information about postal codes |
| INST | Information about Ontario health care institutions funded by the Ministry of Health and Long-Term Care (MOHLTC) | Information about place of delivery and prenatal health care use by the mothers |
| MOMBABY | ICES derived Linked Delivering Mother and Newborns | Pregnancy outcome, gestational age, weight at birth, prematurity, labour instrumentation |
| HYPER | ICES derived Ontario Hypertension Dataset | Information of patients validated to be diagnosed with hypertension |
| ODD | ICES derived Ontario Diabetes Dataset | Information of patients validated to be diagnosed with diabetes |

© 2020 Malic CC et al. *JAMA Network Open.*

**eTable 2.** Diagnostic and Procedural Codes Used in This Study

| ICES variable | Code type | Codes |
|---|---|---|
| Cleft lip diagnosis | ICD-9 | 7491 |
| | ICD-10 | Q36 |
| Cleft palate diagnosis | ICD-9 | 7490 |
| | ICD-10 | Q35 |
| Cleft lip and palate diagnosis | ICD-9 | 7492 |
| | ICD-10 | Q37 |
| Cleft lip surgery | CCP | 337, 986 |
| | CCI | 1YE80LAXXE |
| | OHIP fee code | E501, S013, T523 |
| Cleft palate surgery | CCP | 361, 395 |
| | CCI | 1FB80LAXXE, 1FC80LA |
| | OHIP fee code | S034 |
| Syndromic OFC pathology | ICD-9<br>ICD-10 | 740-759<br>Q00-Q99 |
| Prematurity | ICD-10 | O660, P072, P073 |
| | ICD-10 | O660, P072, P073 |
| Mother's mental health related healthcare use | ICD-9/DSM IV | 291-298, 301-316 |
| | ICD-10 | F04-F07, F09-F25, F28-F34, F38-F45, F48, F50-F55, F59-F66, F68, F69, F80-F84, F88-F95, F98, F99 |
| Mother's history of epilepsy | ICD-9, OHIP diagnostic code | 345 |
| | ICD-10 | G40, G41 |
| Mother's mental health related healthcare visit with primary care provider *(Algorithm: OHIP all mental health codes plus a mental health diagnostic code, or non MH contact either with the primary care provider with a mental health diagnostic code (with OHIP specialist code 00)* | OHIP all mental codes | 290, 295- 298; 300-304: 306; 309; 311, 331, 797, 897-902; 904-906,909 |
| | OHIP general service codes | K005, K007, K623<br><br>A001,3-8;A888;A905-6 |
| Type of prenatal care provided by family physician | OHIP fee code | A005, A006, A665, A920, Q606, Q607 |
| Type of prenatal care provided by family obstetrician | OHIP fee code | P002-P005 |
| | Database: IPDB | GP, OBGYN |
| History gestational diabetes | ICD-9 | 6480 |
| | ICD-10 | O224, O229 |
| Gestational hypertension | ICD-9 | 401, 405, 6420-6429 |
| | ICD-10 | O13-O16, I10, I15 |
| | OHIP diagnostic code | 642 |
| Labour induction | CCI | 5AC30 |
| | CCP | 8501, 851, 855 |
| Instrumentation during delivery | CCI | 5MD53-55, 5MD56PC, PF, PJ |
| | CCP | 84 |
| C-section | CCI | 5MD60 |
| | CCP | 86 |
| | OHIP fee code | P018 |

© 2020 Malic CC et al. *JAMA Network Open.*

**eTable 3.** Comparison of Overall OFC Incidence From FY 1994 to 2017 With the Decade FY 2007-2016 Within Each LHIN

| LHIN # | LHIN name | 2007-2016 (events/ 1000 births) | 1994-2017 (events/ 1000 births | p-value |
|---|---|---|---|---|
| 1 | Erie St. Clair | 1.37 | 1.40 | 0.54 |
| 2 | South West | 1.13 | 1.26 | 0.92 |
| 3 | Waterloo Wellington | 1.03 | 1.17 | 0.80 |
| 4 | Hamilton Niagara Haldimand Brant | 1.30 | 1.32 | 0.54 |
| 5 | Central West | 0.80 | 0.86 | 0.81 |
| 6 | Mississauga Halton | 0.77 | 0.84 | 0.82 |
| 7 | Toronto Central | 0.78 | 0.84 | 0.86 |
| 8 | Central | 0.79 | 0.87 | 0.86 |
| 9 | Central East | 0.97 | 1.11 | 0.99 |
| 10 | South East | 1.04 | 1.12 | 0.63 |
| 11 | Champlain | 1.04 | 0.99 | 0.35 |
| 12 | North Simcoe Muskoka | 1.49 | 1.25 | 0.09 |
| 13 | North East | 1.37 | 1.34 | 0.44 |
| 14 | North West | 1.17 | 1.25 | 0.59 |

© 2020 Malic CC et al. *JAMA Network Open.*

**eTable 4.** Distribution and Timing of Surgical Procedures for Cleft Lip and/or Cleft Palate Repair in the OFC Cohort

| **Variable** | **OFC group (N=3051)** |
|---|---|
| **Children born with Cleft Lip Only, N (%) of cohort** | **595 (19.5)** |
| Male:Female ratio in children born with CL | 1.95 males to 1 female |
| Cleft Lip Surgery in the first 2 years of life | 537 (90.3) |
| Time to CL surgery for CL (days since birth) | |
| Mean ± SD | 142.6 ± 83.9 |
| Median (IQR) | 125 (101-156) |
| **Children born with Cleft Palate Only, N (%) of cohort** | **1345 (44.1)** |
| Male:Female ratio in children born with CP | 0.78 males to 1 female |
| Cleft palate Surgery in the first 2 years of life | 1033 (76.8) |
| Time to CP surgery (days since birth) | |
| Mean ± SD | 396.3 ± 87.4 |
| Median (IQR) | 384 (350-435) |
| **Children born with Cleft Lip and Palate, N (%) of cohort** | **1111 (36.4)** |
| Male:Female ratio in children born with CLP | 1.87 males to 1 female |
| Cleft Lip & Palate surgery in the first 2 years of life | 962 (86.6) |
| Time to CL surgery for CLP patients (days) | |
| Mean ± SD | 145.6 ± 64.8 |
| Median (IQR) | 130 (103-170) |
| Time to CP surgery for CLP patients (days since birth) | |
| Mean ± SD | 346.6 ± 118.8 |
| Median (IQR) | 369 (330-405) |

Note: Orofacial cleft (OFC), Standard deviation (SD)
Note: P-value comparing mean time to cleft palate surgery between cleft palate only group and cleft lip and palate group (ie. 396.3 vs. 346.6) is <0.001.

© 2020 Malic CC et al. *JAMA Network Open.*
